# Medical Opinion and Movement

**Published:** 1906-06-23

**Authors:** 


### Medical Opinion and Movement.

The profession are extremely grateful to His Majesty King Edward for the continued personal interest which he manifests in everything which can tend to promote the adequate treatment and welfare of the sick and suffering. It was evident that both the King and Queen Alexandra had real pleasure in opening the King's Sanatorium, and that they were struck with the magnificence of its situation. How deep is the personal interest and thoughtfulness of His Majesty is proved by the expression of his desire that the King's Sanatorium shall provide for that large class of persons of slender means in professional and other employments, for which no provision for sickness of this kind at present exists. The Queen, too, has shown her deep interest in the fight against tuberculosis by becoming the patron of the Queen Alexandra Sanatorium at Davos. His Majesty has no more loyal subjects than the members of the medical profession.

Some correspondence is proceeding in the press on the subject of the ever-growing cost to the ratepayers of the existing provision for the insane. One correspondent shows that upwards of forty millions of pounds have been expended on the maintenance of lunatics within a comparatively short period of years, and regards such expenditure as wasteful. One remedy is suggested by an American, Mr. Patterson, who has introduced a Bill into the Senate of the United States to establish a laboratory for the study of the criminal, pauper, and defective classes. The Bill proposes that the United States Treasury should appropriate £5,000 a year to defray the working expenses, in addition to the requisite amount of funds for the proper equipment and for carrying on the work of this laboratory. The work is to include not only laboratory investigations, but a collection of sociological and pathological data, especially such as may be found in institutions for the criminal, pauper, and defective classes. Most thoughtful people would welcome an attempt to help the human race by making use of the material such institutions afford, which, though costly to the normal population, is at present utterly wasted. The attempt to find a remedy by closely investigating all the material afforded by such institutions is worthy of all encouragement, though it can hardly hope to escape the assaults of the classes who have little to do, and less business aptitude, but who are apt to wallow in the kind of sentiment which is often but an index to the degeneration of the race.

The provision of nursing homes for paying patients where operations can be performed under the most favourable conditions, and the relations of members of the profession to such homes, are causing criticism of the present methods by the patients and their friends, and some difference of opinion in the medical profession itself. It is held to be an abuse that patients who use such homes are sometimes placed at a disadvantage owing to the connection, direct or indirect, of practitioners and consultants with the homes to which they send patients. The leaders of medical opinion in this country have always been opposed to members of the profession having a pecuniary interest of any kind in such homes, though the practice is common enough in the United States and throughout Europe. No doubt it would be better for the patient that the practitioner should be the declared owner or part owner of a home, for then he could be held responsible when the patient is improperly fed or looked after in any other respect. As matters stand at present a surgeon may dominate one of these homes by filling most of the beds, declaring himself to be not responsible for the management in any way, and so give the patients and their friends the impression, rightly or wrongly, that they have no redress against abuse. Experience shows, however, that the surgeon who dominates the management by making habitual use of the home material to his financial success may thus gain for himself the advantage of proprietorship without incurring any financial or personal liability. This state of affairs causes justifiable grumbling and dissatisfaction, and a remedy for it is demanded in the interests of the general body of practitioners and of the public who resort to nursing homes.

We hope that everyone in a position to instruct the relatively ignorant in the purchase of foods will make themselves familiar with the sources of supply of British and Colonial manufactures, and so pass on their information to the humbler folk who have largely to depend upon preserved food-stuffs. Mr. Gunther, the Chairman of Lemco and Oxo, publicly stated last week that the process of manufacture from beginning to end is controlled by expert chemists, and that every batch of the company's goods are analysed and tested both at the works and in Europe before being issued to the trade for public consumption. The company's policy is to work under the control of independent scientists of the highest reputation, including Sir Henry Roscoe, and the sanitary condition of their factories and the whole process of manufacture left nothing to desire. It is undoubtedly true that we have failed in England to institute adequate measures of protection for the people in regard to American foods of this type by organising an efficient inspection at the port of entry. We are glad to know that the authorities of the port of Manchester have succeeded in obtaining a provisional order to enforce in that port the adequate and thorough inspection of all food-stuffs landed within its precincts every year. Adequate inspection, we fear, is only possible at the packing house where the tinned goods are prepared and encased. At the port of entry all that can be attempted is to throw out all tins which exhibit symptoms of the presence of gas, and this at best affords relatively little protection to the consumer. The War Office is arranging to have inspectors at the packing factory from which the goods are sent. Until the importing houses undertake to guarantee a similar examination at the factory by English inspectors of all tinned goods they offer for British consumption, everybody will be wise to avoid eating American tinned meats of every description.

